# Success of Coenzyme Q10 in Treating Steroid-Resistant Nephrotic Syndrome in Jordan: A Case Series

**DOI:** 10.7759/cureus.83231

**Published:** 2025-04-30

**Authors:** Mahdi Q Frehat, Aghadir Alhadidi, Abdallah Almhairat, lubna Alkhatib, Shawq Al Thaher, Ruba Al Assaf, Moath Al Qawaqenah, Batool Mansour, Faisal Khair

**Affiliations:** 1 Pediatric Nephrology, Jordanian Royal Medical Services, Amman, JOR; 2 Pediatrics, Jordanian Royal Medical Services, Amman, JOR; 3 Neonatology, Jordanian Royal Medical Services, Amman, JOR; 4 Nephrology, Jordanian Royal Medical Services, Amman, JOR; 5 Faculty of Medicine, Yarmouk University, Amman, JOR

**Keywords:** coenzyme q10, end-stage kidney disease, focal segmental glomerulosclerosis, nephrotic syndrome, steroid resistance

## Abstract

Focal segmental glomerulosclerosis (FSGS) is one of the leading causes of primary end-stage kidney disease in the pediatric age group. It is commonly associated with steroid-resistant nephrotic syndrome (SRNS), which ultimately leads to impairment in the function of the glomerular filtration system. Genetic studies have revealed nearly 50 types of gene deficiency disorders linked to the development of both FSGS and SRNS. Among these disorders, primary coenzyme Q10 (CoQ10) deficiency is classified as one of the few types that respond well to treatment. CoQ10 plays a crucial role within the mitochondria, including energy production through the electron transport chain. A network of at least 17 genes is necessary for its synthesis. When mutations occur in the genes responsible for CoQ10 production, a deficiency can develop, leading to mitochondrial dysfunction and reduced cellular energy levels. Since CoQ10 is crucial for mitochondrial function, its deficiency has been recognized as a potential therapeutic target. Increasing evidence suggests that CoQ10 supplementation may provide clinical benefit in treating this condition.

We present three pediatric cases of SRNS that did not respond to standard treatment. Despite kidney biopsies revealing FSGS, conventional therapies proved ineffective. The patients were started on CoQ10 supplementation, which led to a complete resolution of nephrotic syndrome. Their kidney function remained within the normal range during follow-up, and proteinuria remained stable, indicating a sustained therapeutic response.

## Introduction

Nephrotic syndrome is a condition caused by dysfunction of the glomerular capillary barrier, leading to proteinuria, edema, and hypoalbuminemia. It has multiple causes and is a well-known reason for pediatric hospital admissions. Genetic mutations play a significant role in its development, as studies have shown [1,2], explaining the strong genetic basis and why it is important to consider genetic testing when dealing with steroid-resistant nephrotic syndrome (SRNS). Mutations in genes such as WT1, TRPC6, CD2AP, PLCE1, INF2, ACTN4, ITGA3, NPHS1, and NPHS2 are among the most frequently reported [3]. However, multiple studies have concluded that mutations in NPHS2 and WT1 account for about 29% of single-gene mutation causes, ultimately resulting in SRNS [4].

Recent genetic studies have identified mitochondrial disorders as a potential cause of nephrotic syndrome in some cases. Mutations affecting genes responsible for coenzyme Q10 (CoQ10) biosynthesis can lead to its deficiency. For example, in this study [5], mutations involving molecules important in CoQ10 synthesis, such as ADCK4, promoted the development of SRNS. Therefore, considering CoQ10 supplementation is as important as the treatment itself.

CoQ10 is a lipid-like antioxidant involved in various cellular processes, including its critical role in the mitochondrial respiratory chain [6]. Primary CoQ10 deficiency, an autosomal recessive mitochondrial disorder [7], can present with neurological, muscular, and renal manifestations [8]. Studies have shown that oral CoQ10 supplementation in deficient patients leads to clinical improvement, highlighting the importance of early diagnosis.

In this report, we present three cases of SRNS that showed a positive response to CoQ10 supplementation, highlighting its potential benefit in children with SRNS due to NPHS2 mutations and indicating a possible role for mitochondrial-targeted therapy in this group.

## Case presentation

Case 1

In September 2021, a 12-year-old boy was admitted to the Nephrology Department at Queen Rania Hospital for Children, Amman, Jordan, with widespread edema, including periorbital swelling, ascites, and scrotal edema, of an undetermined cause. As seen in Table 1, initial laboratory tests showed significant proteinuria (urine albumin +4), moderate hematuria, and a urine pH of 5.5. He had elevated serum creatinine levels of 1.1 mg/dL, while serum albumin levels were low. Based on these results, he was diagnosed with nephrotic syndrome and began receiving the standard protocol of treatment, which consisted of albumin infusions during hospitalization and corticosteroids (prednisolone 2 mg/kg).

**Table 1:** Admission Vital Signs and Laboratory Results of Patient 1

Laboratory tests	Patient results	Reference range	Vitals
Urine albumin	+4 (>1000 mg/dL)	<0.3 mg/dL	BP: 124/82
Urine pH	5.5	6-7.5	HR: 80 bpm
Serum creatinine	1.1 mg/dL	0.4-0.7 mg/dL	O_2_ Sat: 98%
Serum albumin	20 g/L	34-54 g/L	Temp: 36.2°C

Serial renal ultrasounds revealed normal kidney structure with no signs of congenital defects or any acquired abnormalities, confirming the absence of any structural pathology. The patient had no previous medical or family history of proteinuria or any renal abnormalities. After six weeks of treatment, he experienced a relapse and was diagnosed as a case of SRNS.

This patient underwent two renal biopsies. The first one, performed in September 2022, showed negative immunoreactions, but the second confirmed the presence of focal segmental glomerulosclerosis (FSGS). Meanwhile, the genetic study revealed a homozygous NPHS2 gene mutation.

As part of the treatment, he was given cyclosporine and tacrolimus, both of which were later discontinued due to rising serum creatinine levels. His condition required frequent hospitalizations due to persistent edema and intravascular depletion.

No clinical improvement occurred after additional therapy, which consisted of three rituximab doses, the last of which was administered in September 2024. Moreover, a single plasmapheresis session was attempted, but the treatment was discontinued due to poor tolerance. The patient was started on cyclophosphamide (Endoxan) in September 2024 for one month; however, due to the lack of response, the treatment was discontinued in October 2024.

In October 2024, the patient was started on CoQ10 supplement therapy (a dose of 30 mg/kg/day), which led to significant clinical improvement. Since then, his kidney function test results have remained normal, showing no signs of deterioration, and the patient has not experienced any symptoms or required further hospital admissions. His current maintenance medications include prednisolone (three tablets every other day), mycophenolate mofetil (CellCept), Moduretic, furosemide (Lasix), and CoQ10.

Case 2

In December 2023, a three-year-old girl presented to our hospital with swelling in her lower limbs and ascites, which had developed just a few weeks prior to presentation. As shown in Table 2, initial investigations revealed significant proteinuria (urine albumin +4), marked hematuria, and hypoalbuminemia, with a slight elevation in serum creatinine levels. Moreover, she had elevated triglyceride and low-density lipoprotein (LDL) cholesterol levels. Based on these findings, along with a positive family history of FSGS in a second-degree relative, the patient was diagnosed with nephrotic syndrome.

**Table 2:** Admission Vital Signs and Laboratory Results of Patient 2

Laboratory tests	Patient results	Reference range	Vitals
Urine albumin	+4 (>1000 mg/dL)	<0.3 mg/dL	BP: 118/79
Urine pH	5	6-7.5	HR: 88 bpm
Serum creatinine	1.1 mg/dL	0.3-0.5 mg/dL	O_2_ Sat: 98%
Serum albumin	22 g/L	34-54 g/L	Temp: 36.1°C
Low-density lipoprotein (LDL)	271	50-150 mg/dL	-
High-density lipoprotein (HDL)	46	35-65 mg/dL	-
Cholesterol	402	135-200 mg/dL	-

As is standard for patients with nephrotic syndrome, she was started on prednisolone (2 mg/kg/day) and admitted for albumin infusion. Serial renal ultrasounds showed normal kidney structure without any abnormalities. A renal biopsy was performed, confirming the diagnosis of FSGS. She was initiated on cyclosporine, but it did not produce a significant clinical response, leading to a diagnosis of SRNS. Given the severity of her condition, genetic studies were conducted and identified an NPHS2 mutation, confirming a diagnosis of familial FSGS.

Following this diagnosis, CoQ10 was added to her treatment regimen (in a standard dose of 30 mg/kg/day), which also included cyclosporine, captopril, prednisolone, and atorvastatin (Lipitor). Since starting this combination therapy, the patient has remained clinically stable, with no further episodes of edema.

Case 3

A three-year-old male patient presented to the Emergency Room at Queen Rania Hospital for Children with generalized edema, which had developed two days prior to presentation. Initial laboratory investigations (Table 3) showed significant proteinuria (urine albumin +4), a urine pH of 5, hypoalbuminemia (serum albumin 24 g/L), anemia (Hb 9 g/dL), and elevated serum creatinine levels (2.23 mg/dL). Based on these findings, he was admitted to the hospital with a diagnosis of nephrotic syndrome and started on standard treatment, which included corticosteroids (prednisolone 2 mg/kg/day) along with albumin infusions.

**Table 3:** Admission Vital Signs and Laboratory Results of Patient 3

Laboratory tests	Patient results	Reference range	Vitals
Urine albumin	+4 (>1000 mg/dL)	<0.3 mg/dL	BP: 114/86
Urine pH	5	6-7.5	HR: 110 bpm
Serum creatinine	2.23 mg/dL	0.3-0.5 mg/dL	O_2_ Sat: 96%
Serum albumin	24 g/L	34-54 g/L	Temp: 36.2°C

A detailed medical history revealed that he had been diagnosed with SRNS at the age of two, following a relapse despite receiving standard nephrotic syndrome treatment. His medication history showed that he was maintained on thyroxine and calcium carbonate. There was no family history of renal disease.

Serial kidney ultrasounds showed diffusely hyperechoic kidneys with loss of corticomedullary differentiation, but no congenital malformations or structural abnormalities. A kidney biopsy confirmed the presence of FSGS. He was initially started on cyclosporine (Neoral) alongside prednisolone, but the treatment was discontinued due to the development of acute kidney injury (AKI).

Given his clinical course, a genetic study was performed and identified a homozygous NPHS2 gene mutation, which explained his presentation. A trial of CoQ10 was introduced alongside antihypertensive medications and thyroxine. Since starting this regimen, the patient has remained stable and has experienced significantly fewer relapses.

Pathology and molecular findings

A renal biopsy performed on Patient 1 revealed global and segmental sclerosis. The remaining viable glomeruli appeared normal, with no signs of mesangial hypercellularity, necrotizing lesions, or hyaline globules. The blood vessels, tubulointerstitial compartment, and glomerular capillary basement membrane were unremarkable. Electron microscopy showed focal effacement of podocyte foot processes (approximately 40%), with no electron-dense deposits or abnormalities in the tubulointerstitial structures.

Patient 2’s renal biopsy demonstrated diffuse effacement, sclerosis, and detachment of the epithelial foot processes. These findings were consistent with a diagnosis of FSGS.

The biopsy for Patient 3 consisted of two renal core samples containing 35 glomeruli, analyzed using hematoxylin and eosin (H&E), reticulin, Masson’s trichrome (MT), and periodic acid-Schiff (PAS) stains. Seven glomeruli exhibited segmental sclerosis with adhesions involving the tip and hilar regions, highlighted by PAS staining. The remaining glomeruli appeared within normal limits. The affected glomeruli accounted for 20% of the total sample. Tubular findings included focal variations in size and the presence of a few intratubular casts. The interstitium showed mild chronic inflammation, while the blood vessels were within normal limits. The overall findings were consistent with FSGS. A summary of the clinical and diagnostic features across the three SRNS cases treated with CoQ10 is provided in Table 4, presented in the Appendix.

**Figure 1:**
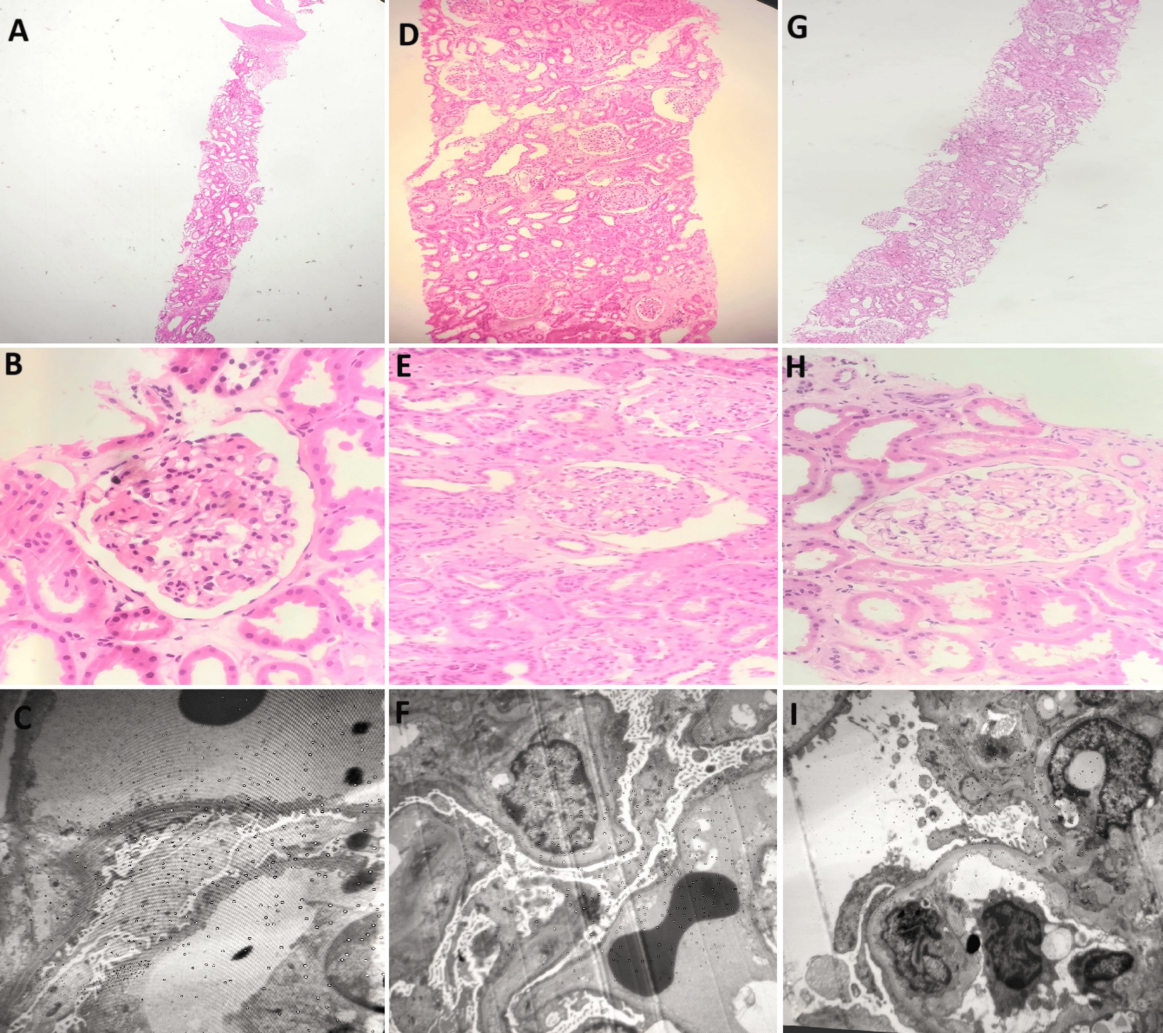
Biopsies of Three Patients Patient 1, (A-C): Renal biopsy showed global and segmental sclerosis, with otherwise normal glomeruli. Electron microscopy (EM) revealed approximately 40% podocyte foot process effacement, without deposits. Patient 2, (D-F): Renal biopsy showed diffuse foot process effacement and sclerosis, consistent with focal segmental glomerulosclerosis (FSGS). Patient 3, (G-I): Biopsy showed segmental sclerosis with tip and hilar adhesions (20% of glomeruli), mild tubular changes, and chronic inflammation - consistent with FSGS.

Genetic testing was conducted for Patients 1-3 to investigate potential genetic factors. All were found to have a homozygous NPHS2 gene mutation. This mutation follows an autosomal recessive inheritance pattern and is a well-known cause of nephrotic syndrome. Additionally, NPHS2 mutations have been associated with impaired CoQ10 functionality (Figure 1).

## Discussion

SRNS is a renal disorder that often leads to kidney failure. Recent studies have highlighted primary CoQ10 deficiency, which results from genetic mutations in the enzymes responsible for CoQ10 production. For instance, previous studies [9,10] revealed that mutations in CoQ6 and CoQ2, which are critical in the biosynthesis of CoQ10, have led to the development of SRNS along with extrarenal manifestations, highlighting the close association between mitochondrial dysfunction and SRNS. Consequently, CoQ10 supplementation should be considered in the early treatment of SRNS. These studies underscore the importance of early CoQ10 supplementation in the management of SRNS [11,12].

As we know, CoQ10 is essential for proper mitochondrial function. A study by Quinzii and Hirano in 2010 discusses the role of CoQ10 in mitochondrial function, revealing that its deficiency has led to the development of multiple mitochondrial disorders, including renal manifestations [13]. Another case report from 2006, by Lopez et al., further supports the role of mitochondrial dysfunction in the development of SRNS [14]. They reported a patient with Leigh syndrome, nephrotic syndrome, and CoQ10 deficiency due to a mutation in the PDSS2 gene - the first enzyme in the CoQ10 biosynthetic pathway.

In December 2024, a case study was published describing a compound heterozygous mutation in the PDSS1 gene in a three-year-old female patient, resulting in CoQ10 insufficiency. This patient experienced developmental regression and eventually developed SRNS. Despite being treated with high-dose CoQ10 supplementation after the disease had advanced, there was only slight improvement, suggesting that early detection and intervention are crucial for better outcomes [15]. On the other hand, some studies have shown promising results with CoQ10 supplementation in patients with primary CoQ10 deficiency. A review found that early treatment with high-dose CoQ10 in patients with mutations affecting CoQ10 biosynthesis could yield positive responses [16,17].

Although specific case reports from Jordan are not readily available, the global evidence emphasizes the importance of considering genetic testing for CoQ10 biosynthesis defects in patients with SRNS. Early diagnosis and prompt treatment with CoQ10 supplementation could provide an effective therapeutic option for these patients.

## Conclusions

SRNS is a serious clinical challenge, often leading to permanent kidney damage and requiring intensive immunosuppressive therapy. Recent data have highlighted mitochondrial dysfunction, specifically CoQ10 deficiency, in the development of SRNS. In this paper, we report three pediatric cases of SRNS in which patients experienced significant clinical improvement and long-term remission of proteinuria after being treated with CoQ10 supplements. These results support the potential of CoQ10 as a targeted treatment option for certain cases of SRNS, particularly in patients with genetic mutations affecting CoQ10 production. This suggests that early genetic screening for mitochondrial-related nephrotic syndromes could enable timely intervention, ultimately improving long-term kidney function. However, additional research is needed to develop standardized guidelines for CoQ10 supplementation in the management of SRNS.

## Appendices

**Table 4:** Summary of Clinical and Diagnostic Features Across Three SRNS Cases Treated With CoQ10 FSGS, Focal segmental glomerulosclerosis; SRNS, Steroid-resistant nephrotic syndrome; CoQ10, Coenzyme Q10

Parameter	Patient 1	Patient 2	Patient 3
Age/sex	12 years/male	3 years/female	3 years/male
Initial presentation	Generalized edema, ascites, scrotal swelling	Lower limb edema, ascites	Generalized edema
Urine albumin	+4 (>1000 mg/dL)	+4 (>1000 mg/dL)	+4 (>1000 mg/dL)
Serum creatinine	1.1 mg/dL	1.1 mg/dL	2.23 mg/dL
Serum albumin	20 g/L	22 g/L	24 g/L
Renal biopsy findings	FSGS with global and segmental sclerosis; ~40% podocyte effacement	FSGS with diffuse sclerosis and foot process effacement	FSGS with tip/hilar sclerosis (20% glomeruli), mild tubular changes
Genetic mutation	Homozygous NPHS2	Homozygous NPHS2	Homozygous NPHS2
Previous treatments	Prednisolone, Cyclosporine, Tacrolimus, Rituximab, Cyclophosphamide	Prednisolone, Cyclosporine	Prednisolone, Cyclosporine
SRNS diagnosis basis	Non-response to corticosteroids and immunosuppressants	Lack of response to steroids and cyclosporine	Relapse despite standard therapy; resistant to cyclosporine
CoQ10 initiation	October 2024 (after failure of the conventional management)	Post-genetic confirmation	Post-biopsy/genetic confirmation
CoQ10 concurrent medications	Prednisolone, Mycophenolate, Moduretic, Lasix	Prednisolone, Cyclosporine, Captopril, Atorvastatin	Prednisolone, Antihypertensives, Thyroxine
Response to CoQ10	Complete remission; no relapse or hospital readmission	Clinically stable, no further edema	Fewer relapses; improved clinical stability
